# Early Efficacy Assessment of Arthroscopic Lower Trapezius Transfer With Tendon Autograft in the Management of Massive Irreparable Posterosuperior Rotator Cuff Tears

**DOI:** 10.3389/fsurg.2021.796359

**Published:** 2022-01-07

**Authors:** Lingchao Ye, Dawei Han, Qingguo Zhang, Xiangdong Yang, Tao-Hsin Tung, Xiaobo Zhou

**Affiliations:** ^1^Department of Sports Medicine, Taizhou Hospital of Zhejiang Province Affiliated to Wenzhou Medical University, Linhai, China; ^2^Public Laboratory, Evidence-Based Medicine Center, Taizhou Hospital of Zhejiang Province Affiliated to Wenzhou Medical University, Linhai, China

**Keywords:** massive irreparable rotator cuff tears, lower trapezius transfer, tendon autograft, rotator cuff repair, arthroscopic

## Abstract

**Objectives:** To explore the indications and surgical techniques for arthroscopic lower trapezius transfer (LTT) with tendon autograft in managing massive irreparable posterosuperior rotator cuff tears (PSRCTs); to validate the feasibility, safety, and efficacy of this technique.

**Methods:** This study retrospectively enrolled 23 patients with massive irreparable PSRCTs, admitted to and followed up by the Taizhou Hospital of Zhejiang province between July 2020 and April 2021, and treated with ipsilateral LTT and ipsilateral hamstring tendon autograft. The control group consisted of 23 patients with massive RCTs receiving conventional repair procedures within the same frame. Follow-up data at the preoperative visit, and postoperative month 3 were collected to assess the active range of motion, Constant–Murley Score (CMS),American Shoulder and Elbow Surgeons Standardized Shoulder Assessment Form (ASES), University of California, Los Angeles (UCLA)shoulder score, visual analog scale (VAS)and the post-operative MRI results, all of which could provide a comprehensive postoperative early efficacy assessment.

**Results:** Three months follow-up visits were completed for all patients, revealing improvements in all aspects compared to the preoperative state, with no complications, such as postoperative infection of surgical sites and nerve injuries of infection and nerve injury. The distribution of active shoulder range of motion of patients and function scores with two types of operation was as follow: angles of flexion and lifting (130.00° ± 31.55° vs. 90.78° ± 19.85°), abduction (123.26° ± 30.47°vs. 85.87° ± 18.74°), external rotation at side (101.74° ± 14.74° vs. 91.74° ± 11.92°), external rotation at 90° abduction (41.52° ± 21.97° vs. 24.57° ±12.60°), VAS (0.74± 0.81 vs. 1.87 ±0.87), CMS(56.3 ± 13.01 vs. 48.30 ± 8.38), UCLA shoulder score (24.04 ± 2.88 vs.20.96 ± 3.47), ASES (72.91 ± 9.99 vs.60.74 ± 8.84). Significantly better improvements were found in the study group on month 3.19 of 23 patients in the study group and 17 of 23 patients in the control group underwent MRI on the 3 months follow up. Retear was found in only one patient who had grade 4 subscapularis tendon injury, However, revision was not performed due to postoperative pain relief and functional improvement.

**Conclusion:** Compared to conventional repair procedures, in the early postoperative period, LTT with tendon autograft could achieve better pain relief, more rapid motor functional recovery, and higher functional scores for massive irreparable PSRCTs.

## Introduction

In the 21st century, with the accelerated aging of the Chinese population, cases of rotator cuff injuries are also increasing, as with patients with irreparable rotator cuff tears (RCTs). The pain and functional limitations of irreparable RCTs seriously impair the quality of life. Massive Irreparable Posterosuperior Rotator Cuff Tears is internationally defined as: after removal of avascular tissue, when placing the upper arm in the lateral receptor, the tendon tissue is so poor that no tendon-bone repair cannot be performed directly (1, 2). Various treatments have been developed for massive RCTs, including conservative treatment, arthroscopic debridement, transfer and fixation of the long head of biceps, superior capsular reconstruction (SCR), latissimus dorsi transfer, lower trapezius transfer (LTT), subacromial balloon spacer implantation, and reverse shoulder replacement (3, 4). The selection of procedures and their efficacies have been questioned by many experts due to the high failure rate of the massive RCT repair procedures, which is probably caused by intramuscular fat infiltration and severe atrophy of the rotator cuff resulting from the chronic course of the disease. Therefore, the treatment of massive RCTs is a great challenge in the clinical practice of orthopedics and sports medicine (5). As for the treatment of irreparable posterosuperior rotator cuff tears, an increasing number of studies focused on latissimus dorsi transfer (LDT) and lower trapezius transfer (LTT), as well as their efficacy in comparison to one another, with results showing that LTT possessed advantages such as better aid in synergistic movement, application of force on humeral head in a direction more consistent with infraspinatus muscle, etc. However, the length of lower trapezius muscle limited the feasibility of LTT alone, and achilles tendon allografts bridging technique was proposed to address this issue, as reported in previous studies, Ideal outcomes were observed in cadaver studies, open surgeries, and arthroscopic surgeries (6–8). This study explored attempts of LTT with tendon autograft in the treatment of massive irreparable PSRCTs and summarized clinical follow-up data.

## Methods

### General Characteristics

**Figure 1:**
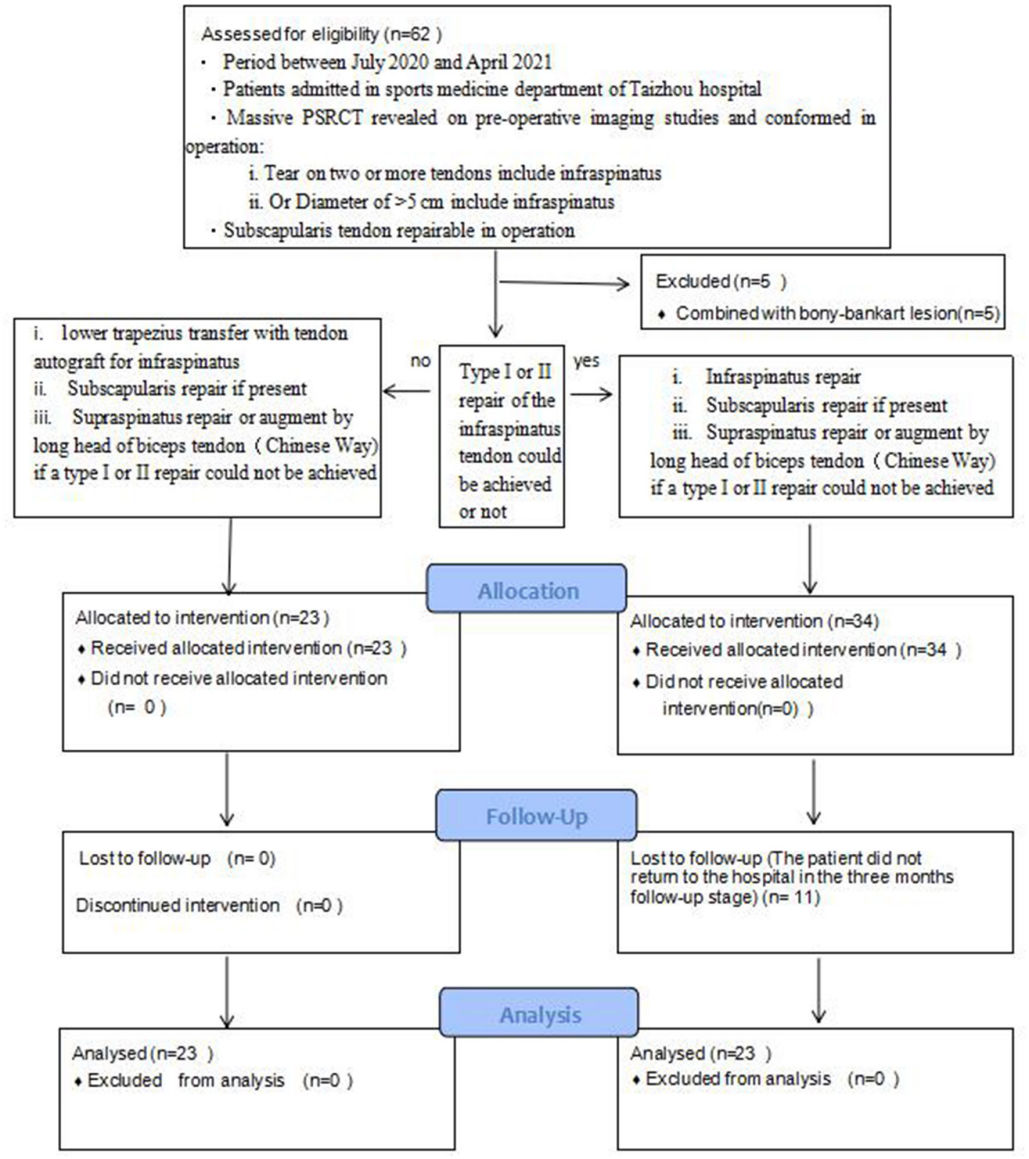
Flowchart showing patient inclusion and exclusion.

A complete ethics review was conducted for every case by the Ethics Committee of Taizhou Hospital, and approval was obtained. This study included 23 patients (seven men and 16 women; mean age = 63.61 years) with massive irreparable PSRCTs receiving ipsilateral LTT with ipsilateral hamstring tendon autograft between July 2020 and April 2021 in the study group. Another 23 patients (eight men and 15 women; mean age = 63.22 years) who underwent conventional repair procedures within the same time frame were enrolled as the control group. The flowchart of patient inclusion and exclusion is shown in Figure 1.

### Inclusion Criteria for the Study Group

Massive PSRCT revealed on pre-operative imaging studies and conformed in operation:

i Tear on two or more tendons include infraspinatus
ii Or Diameter of >5 cm include infraspinatus

Complicated with reparable subscapularis tendon injury confirmed by arthroscopy

Type I or II repair (9) of the infraspinatus tendon could not achieved.

### Inclusion Criteria for the Control Group

Patients who met the following criteria were used as controls:

Massive PSRCT revealed on pre-operative imaging studies and conformed in operation:

i Tear on two or more tendons include infraspinatus
ii Or Diameter of >5 cm include infraspinatus

Complicated with reparable subscapularis tendon injury confirmed by arthroscopy

Type I or II repair (9) of the infraspinatus tendon could be achieved.

### Exclusion Criteria

1) Osteoarthritis of Hamada grade 5 revealed by pre-operative imaging.
2) Pigmented villonodular synovitis.
3) Definitively diagnosed glenohumeral instability or axillary nerve palsy.
4) Concurrent shoulder dislocation or acromioclavicular dislocation.
5) Rheumatic arthritis or rheumatoid arthritis.
6) History of other shoulder surgery or joint infection.

### Surgical Technique

#### Surgical Technique in the Study Group

**Figure 2:**
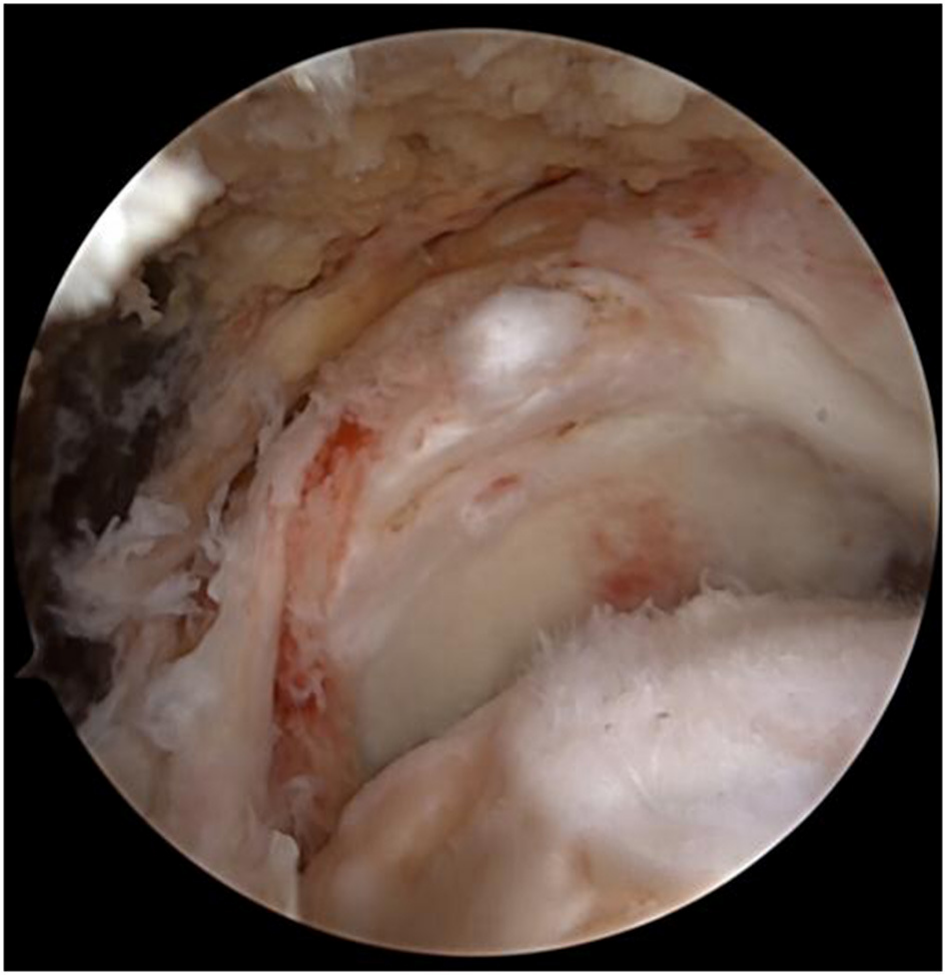
The infraspinatus tendon could not be pulled back into the footprint area.

The lateral decubitus position was assumed, with the patient leaning back at 45° and shoulder joint at 45° abduction and 15° flexion. A traction of 4 kg was applied to the shoulder joint. Standard anterior and posterior incisions for arthroscopy, anterolateral acromial incision, lateral mid-acromial incision, and anterior acromial incision were performed, approximately 1.0 cm each. Tears of the supraspinatus and infraspinatus tendons at the insertion sites were identified under arthroscopy. The infraspinatus tendon could not be pulled back into the footprint area (Figure 2).

**Figure 3:**
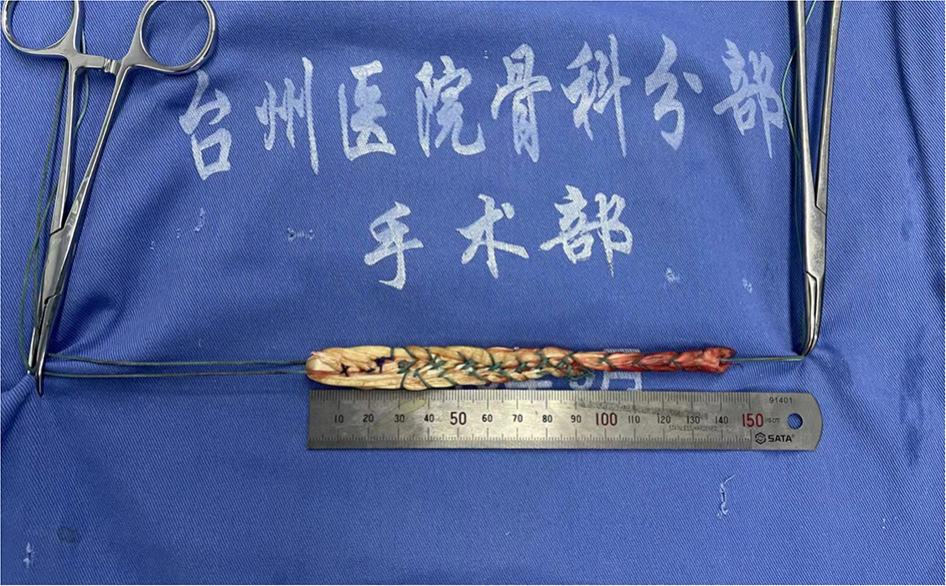
Hamstring tendon of the four strands were stitched into a wide, flat shape.

A transverse-oblique incision of 2–3 cm was made at the interior inferior margin of the ipsilateral tibial tubercle, and the semitendinosus tendon and gracilis tendon were extracted as autografts and folded in half to form a loop. A No. 5 Ethibond suture was passed through the loop. The free ends of the autograft were stitched together. The autograft was folded in unequal lengths to ensure a final length of at least 14 cm (10), in this case, the end closer to the tibia, which was the stronger end, should be on the longer side while folding. The segments of the four strands were stitched into a wide, flat shape (Figure 3).

The subacromial region was debrided using the regular technique, and hyperplastic osteophytes in the anterolateral region of the acromion were removed while maintaining the continuity of the coracoacromial ligament. The insertion sites of the supraspinatus and infraspinatus tendon were polished and freshened. An anchor was fixed at the insertion site of the supraspinatus tendon. The supraspinatus or subscapularis were repaired using regular techniques (an additional anchor might be needed for Subscapularis injury). If the supraspinatus muscle could not be pulled back to the footprint area, biceps tendon transfer and anterosuperior augmentation at the insertion site of the supraspinatus tendon were performed. Another anchor was fixed between the upper margin of the pectoralis major muscle and the exit of the biceps pulley. Meanwhile, the biceps tendon was fixed with one suture, and the other suture was left for further procedures.

**Figure 4:**
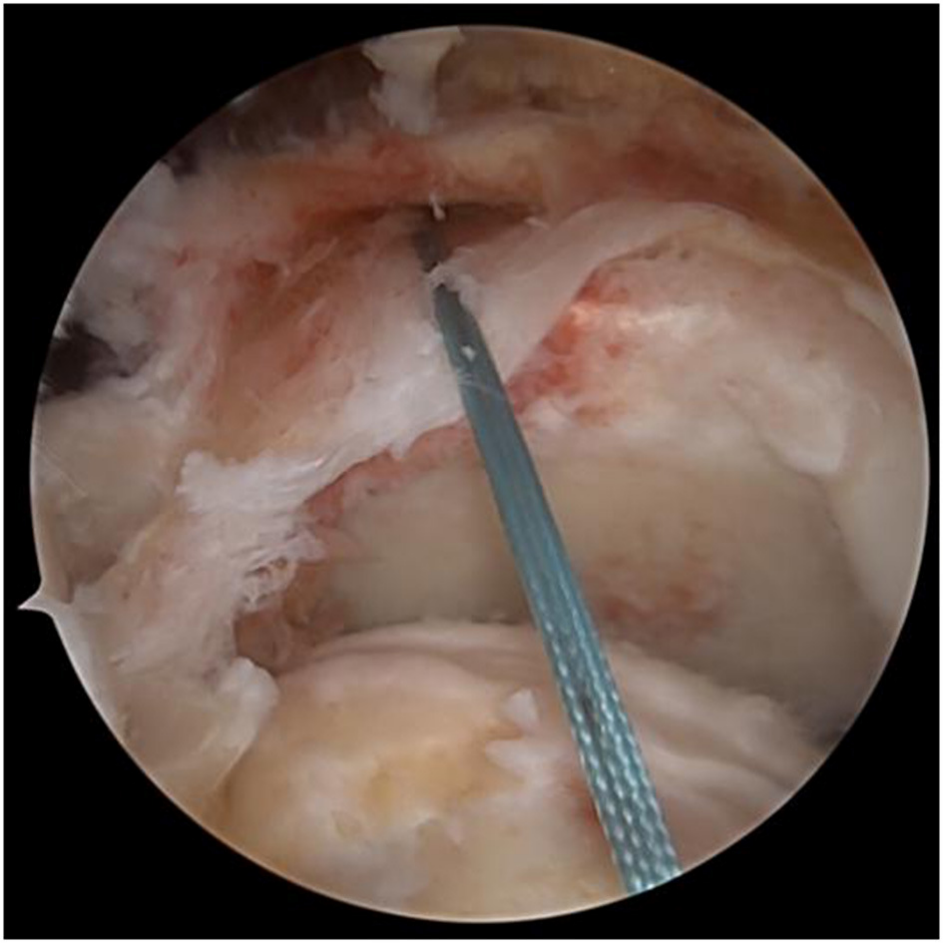
The tendon autograft was thereby introduced into the shoulder joint from the posterior side.

**Figure 5:**
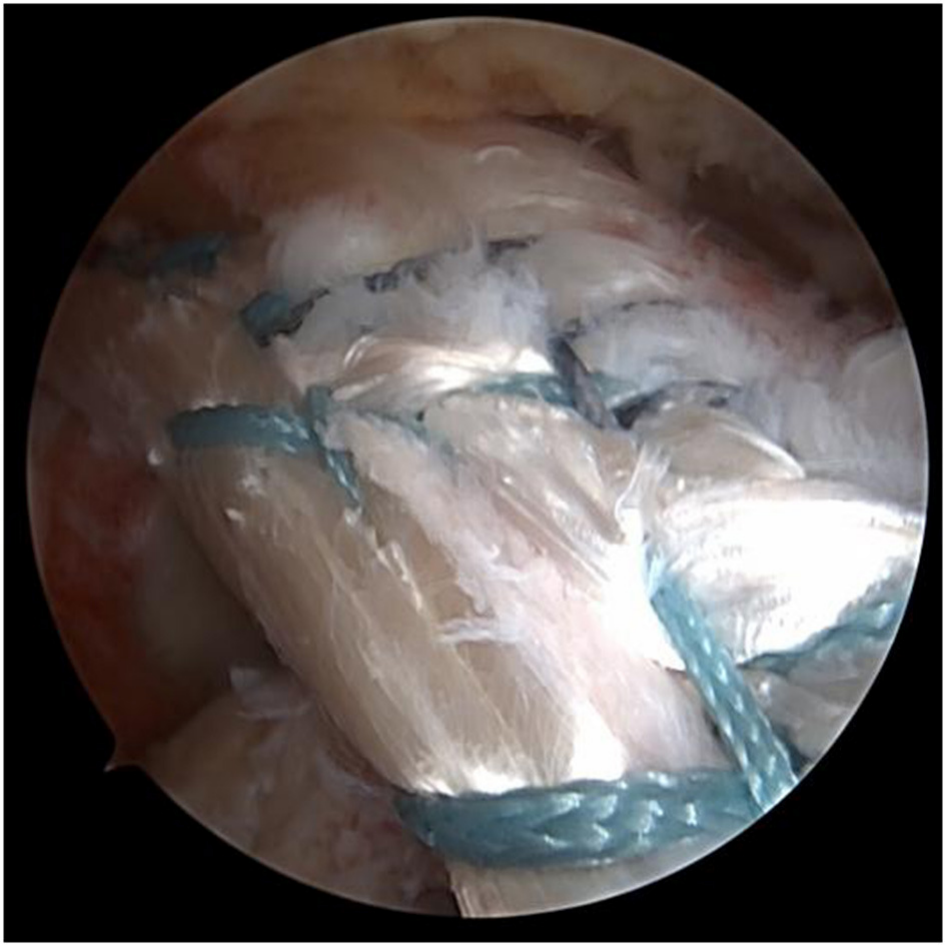
The loop end of the tendon autograft was tied to the anchor fixating the biceps tendon by the suture left from the last step.

**Figure 6:**
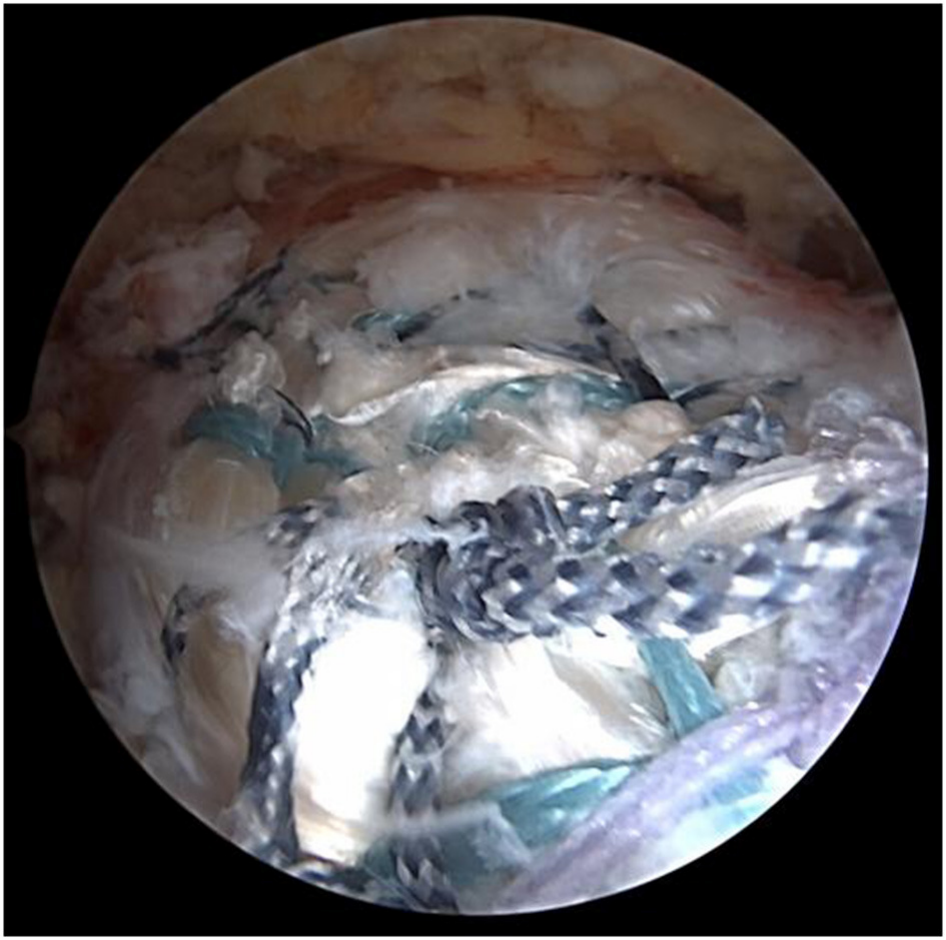
The inner margin of the supraspinatus tendon and the tendon autograft were stitched together side by side if tension was allowed.

**Figure 7:**
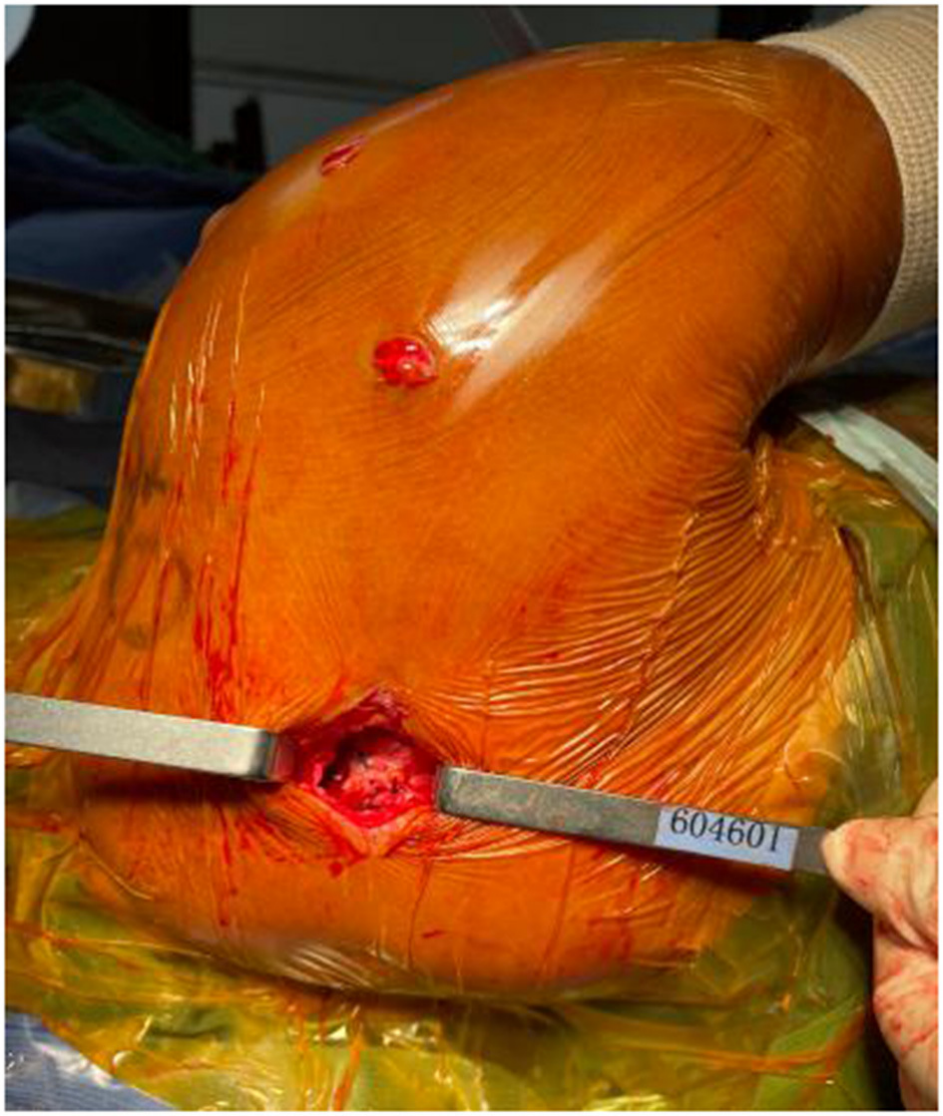
The insertion site of the lower trapezius on the scapular spine was exposed through the posterior scapular incision, dissected, pulled distally, and the free end of the tendon autograft passed across the lower trapezius and stitched.

**Figure 8:**
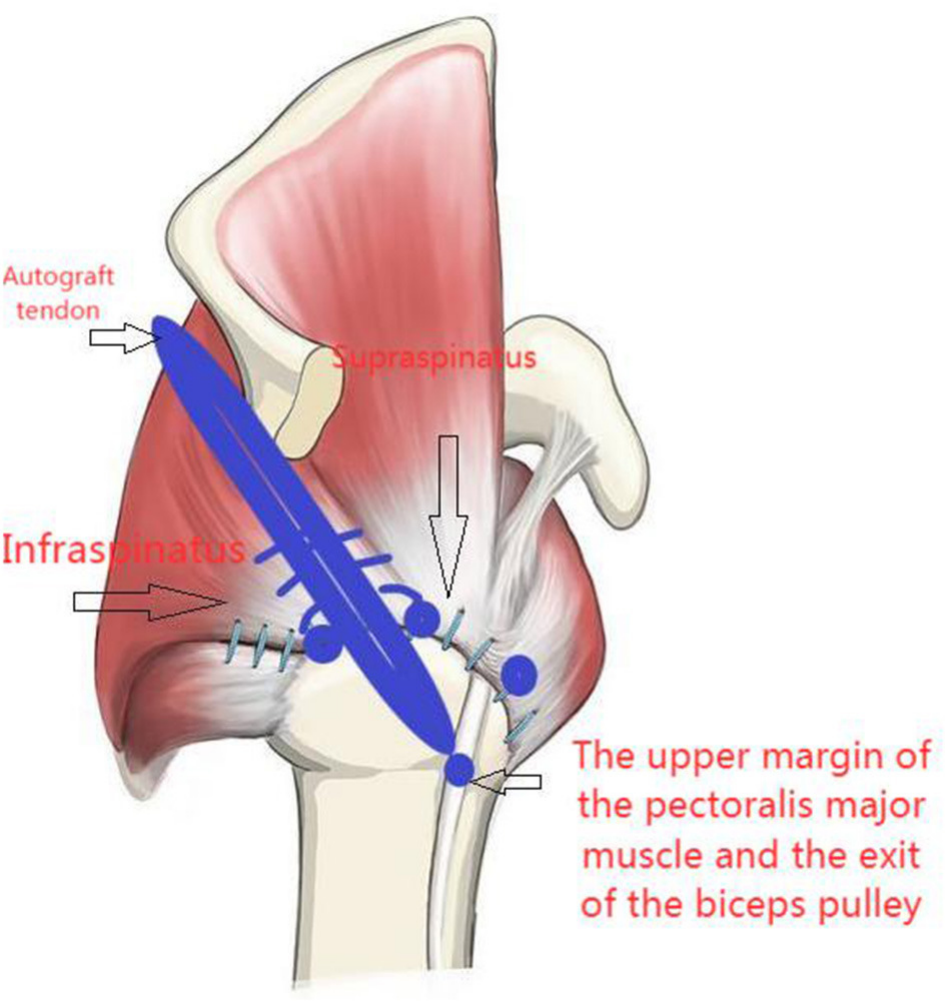
Surgery diagram.

**Figure 9:**
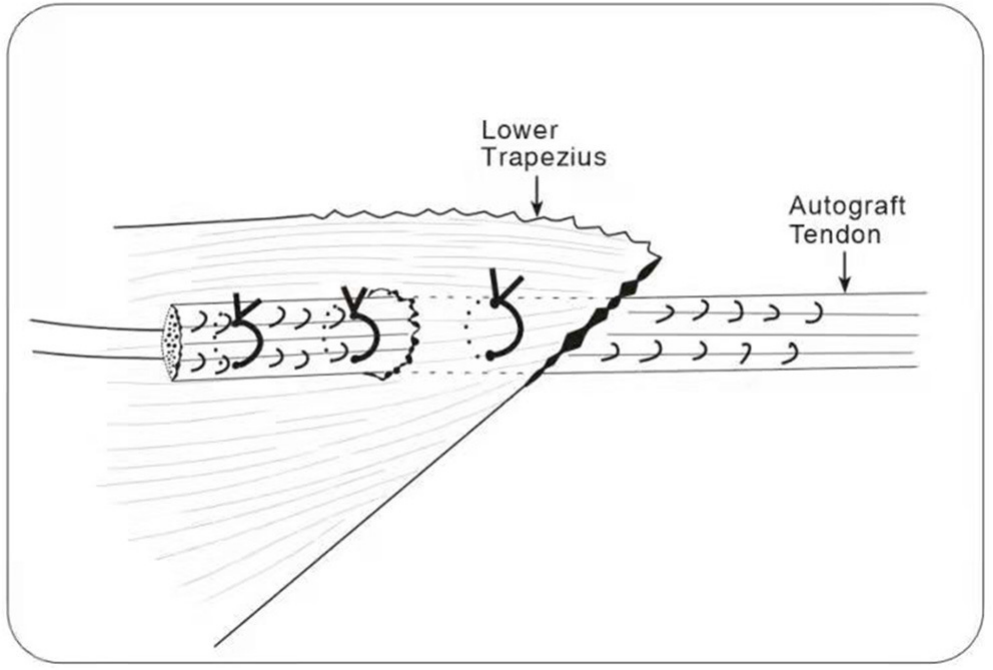
Schematic representation of autograft fixation to the lower trapezius.

A switching stick was inserted via the anterolateral incision under arthroscopy and passed between the two layers of the infraspinatus tendon and near the lower side of the scapular spine, reaching the subcutaneous layer. A 4-cm transverse incision was made at the tip of the switching stick, and the suture on the tendon autograft was held by a suture fetcher and passed through the same tunnel. The tendon autograft was thereby introduced into the shoulder joint from the posterior side, passing through the insertion site of the infraspinatus and greater tubercle. The loop end of the tendon autograft was tied to the anchor fixating the biceps tendon by the suture left from the last step (Figures 4, 5). An anchor was fixated at the footprint area of the infraspinatus tendon, with one suture fixing the graft and another one suturing together the retracted infraspinatus tendon and the tendon autograft. The inner margin of the supraspinatus tendon and the tendon autograft were stitched together side by side if tension was allowed (Figure 6). After fixation under arthroscopy, the insertion site of the lower trapezius on the scapular spine was exposed through the posterior scapular incision, dissected, pulled distally, and passed across and stitched to the free end of the tendon autograft. Subsequently, to prevent insufficient autograft tendon tension, traction on the autograft tendon was applied by attaching weight until fixation inside the shoulder joint was completed, the tension on the autograft tendon was assessed under arthroscopy while abducting and adduction the shoulder joint, and the free ends of the autograft were then crossed through the lower trapezius muscle and stitched above tithe LTT procedure was then concluded (Figures 7–9).

#### Surgical Technique in the Control Group

Patient body position and arthroscopic approach were identical with the study group. Tear of supraspinatus and infraspinatus insertions was confirmed under arthroscopy, and it was deemed possible to pull infraspinatus back to the footprint area with or without the help of myotenolysis, despite some tension, Type I or II repair of the infraspinatus muscle could be achieved. Supraspinatus could be repaired or augment by long head of biceps tendon (Chinese Way), if type I or II repair of the supraspinatu could not be achieved. The subscapularis were repaired using regular techniques (an additional anchor might be needed for some injuries). The order and technique of reparation were designed based on the orientation and type of the tear. Anchors were then fixated and the rotator cuff was repaired with either single or double row suture. Abduction brace was employed for post-operative fixation.

### Postoperative Rehabilitation

All patients had shoulder sling with an abduction pillow after the arthroscopic procedure. From postoperative day 2, joint movement below the elbow was allowed, as well as active shrugging movements and scapular adduction. Movements of the shoulder joint were allowed unless the pain was elicited. At postoperative week 0–3, passive flexion and lifting within 0–60°. Anterior flexion and abduction movements were limited within 60–90° during weeks 3–6. The abduction pillow was removed at postoperative week 6. Then, movements could be allowed within 120° during weeks 6–10. Free movement resumed after week 10. Follow-up visits were scheduled at postoperative weeks 3 and 6, and postoperative month 3, 6, and 12, when pain, range of motion and functional scores were assessed.

### Outcome Measures

We used standard shoulder outcome measures to evaluate the patients, including pain level, activity degree, Constant–Murley Score (11), University of California, Los Angeles(UCLA)shoulder score, and American Shoulder and Elbow Surgeons Standardized Shoulder Assessment Form (ASES). We quantified pain using VAS and measured the range of motion using a goniometer.

### Statistical Analysis

The categorical variables and continuous variables were reported as number (%) or mean ± SD. The two-sample independent t-test and chi-square method were adopted to assess difference in the mean value of continuous variables and distribution of categorical variables between study and control group. The analysis of covariance (ANCOVA) performed to clarify the results of the activity degrees and functional scores between the study and control groups after adjustment for information before surgery. Statistical significance was set at *P* < 0.05 (two-sided). All statistical analyses were performed using IBM SPSS Statistics, version 20 (IBM, Armonk, NY, USA).

## Results

### Baseline Characteristics and Comorbidities

All patients received follow-up care for 3 months. In the study group, 17 (73.9%) patients also underwent long head of biceps tendon transfer and augment (Chinese Way procedure) (12), while only nine (39.1%) in the control group underwent this procedure. Healing of the incision was observed in all patients in the study group, and no obvious complications or adverse events were reported, such as postoperative adhesion or infection in the joint, and dislocation or extrusion of the anchor. In the control group, healing of the incision was also observed, although five patients experienced postoperative intra-articular adhesion and two reported adverse events of intermittent cramping. The demographic and baseline health characteristics are summarized in Table 1. The significant difference between study and control group included operation time, Hamada grade, Supraspinatus fatty infiltration grade, Infraspinatus fatty infiltration grade, Supraspinatus atrophy, Infraspinatus atrophy, Retraction (Patte) supraspinatus and Retraction (Patte) infraspinatus.

**Table 1:** Basic information of patients and operation data statistics.

**Variable**	**Study group mean (SD) or n (%)**	**Control group mean (SD) or n (%)**	**P-value for** ***t*****-test or χ^2^-test**
Age (yr)	63.61 (8.02)	63.17(8.32)	0.86
Height (cm)	155.83 (6.09)	159.39 (7.05)	0.07
Weight (kg)	58.73 (6.33)	60.74 (8.02)	0.35
Female sex	16 (69.6)	15(65.2)	0.52
Dominant arm	21 (91.3)	21 (91.3)	1.00
Operation time (min)	118.61 (35.50)	49.7 (20.48)	<0.001
Length of stay (day)	7.35 (4.58)	6.00 (2.20)	0.21
Injury type			0.92
1. Falling and bumping	11 (47.8)	10 (43.5)	
2. car accident	4 (17.4)	5 (21.7)	
3. others	8 (34.8)	8 (34.8)	
Hamada grade			0.003
• Grade 1	3 (13.0)	15 (65.2)	
• Grade 2	18 (78.3)	8 (34.8)	
• Grade 3	1 (4.3)	0 (0.0)	
• Grade 4	1 (4.3)	0 (0.0)	
Supraspinatus fatty infiltration grade			0.012
• Grade 1	7 (30.4)	18 (78.3)	
• Grade 2	7 (30.4)	3 (13.0)	
• Grade 3	8 (34.8)	2 (8.7)	
• Grade 4	1 (4.3)	0 (0.00)	
Infraspinatus fatty infiltration grade			<0.001
• Grade 1	5 (21.8)	17 (73.9)	
• Grade 2	9 (39.1)	6 (26.1)	
• Grade 3	9 (39.1)	0 (0.0)	
Supraspinatus atrophy			<0.001
• Grade 1	1 (4.3)	8 (34.8)	
• Grade 2	4 (17.4)	12 (52.2)	
• Grade 3	18 (78.3)	3 (13.0)	
Infraspinatus atrophy			<0.001
• Grade 1	2 (8.7)	12 (52.2)	
• Grade 2	6 (26.1)	11 (47.8)	
• Grade 3	15 (65.2)	0 (0.0)	
Retraction (Patte) supraspinatus			0.001
• Grade 1	0 (0.00)	0 (0.0)	
• Grade 2	1 (4.3)	11 (47.8)	
• Grade 3	22 (95.7)	12 (52.2)	
Retraction (Patte) infraspinatus			<0.001
• Grade 1	0 (0.00)	3 (13.0)	
• Grade 2	1 (4.3)	16 (69.6)	
• Grade 3	22 (95.7)	4 (17.4)	

Three months after the operation angles of flexion and lifting (130.00 ± 31.55° vs. 90.78 ± 19.85°), abduction (123.26 ± 30.47°vs. 85.87 ± 18.74°), external rotation at side (101.74 ± 14.74° vs. 91.74 ± 11.92°), external rotation at 90° abduction (41.52 ± 21.97° vs. 24.57 ±12.60°), activity degrees of the study group were found in the study group more than in the control group (*P* < 0.05); VAS in 3 months after surgery (0.74 ± 0.81 vs. 1.87 ± 0.87), CMS (56.3 ± 13.01 vs. 48.30 ± 8.38), UCLA shoulder score (24.04 ± 2.88 vs. 20.96 ± 3.47), ASES (72.91 ± 9.99 vs.60.74 ± 8.84).Better improvements in the CMS, UCLA shoulder score, and ASES were also observed in the study group compared to the control group (*P* < 0.05, Table 2).

**Table 2:** The comparisons of clinical outcomes between study and control groups.

**Variables**	**Mean ± SD**	***P*** **-value for ANCOVA**
Angles of flexion and lifting		0.005
• Study Group:	130.00°± 31.55°	
• Control Group:	90.78°± 19.85°	
Abduction		0.001
• Study Group:	123.26°± 30.47°	
• Control Group:	85.87°± 18.74°	
External rotation at side		0.015
• Study Group:	101.74°± 14.74°	
• Control Group:	91.74°± 11.92°	
External rotation at 90° abduction		0.011
• Study Group:	41.52°± 21.97°	
• Control Group:	24.57°± 12.60°	
VAS in three months after surgery		<0. 0001
• Study Group:	0.74 ± 0.81	
• Control Group:	1.87 ± 0.87	
Constant-Murley in three months after surgery		0.039
• Study Group:	56.3 ± 13.01	
• Control Group:	48.30 ± 8.38	
UCLA in three months after surgery		0.004
• Study Group:	24.04 ± 2.88	
• Control Group:	20.96 ± 3.47	
ASES in three months after surgery		<0. 0001
• Study Group:	72.91 ± 9.99	
• Control Group:	60.74 ± 8.84	

### Imaging Assessment

**Figure 10:**
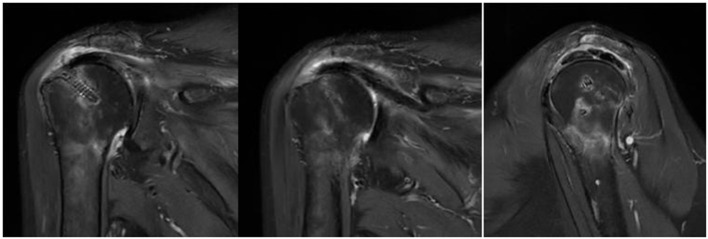
Sugaya's classification types I and II in postoperative MRI.

**Figure 11:**
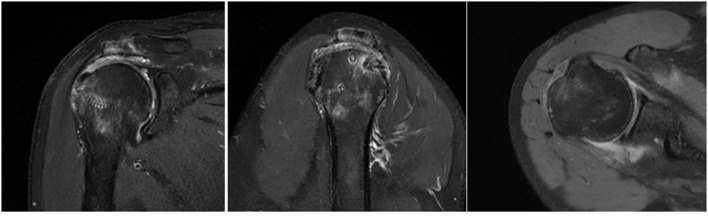
Sugaya's classification type III in postoperative MRI.

**Figure 12:**
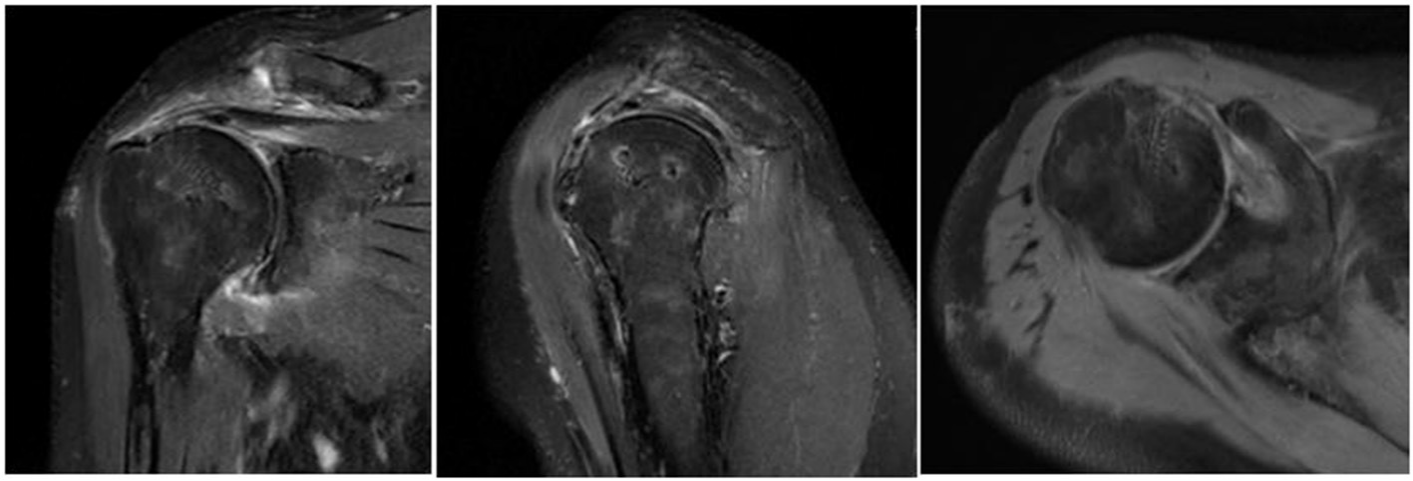
Postoperative MRI images of the control patient.

Nineteen of twenty three patients in the study group and 17 of 23 patients in the control group underwent magnetic resonance imaging (MRI) assessment on three month follow-up. The Sugaya's types I and II (Figure 10) healing in the supraspinatus and infraspinatus were compared between the two group. For infraspinatus, 18 of 19 (94.7%) in the study group VS 17 of 17 (100%) in the control group and 13 of 19(68.4%) VS 15 of 17 (88.2%) as to the supraspinatus. The percentage of healing was a little bit high in the control group but there was no significant difference between them *p* = 0.314 and *p* = 0.288. One patient in the study group experienced retear, who had a grade 4 subscapularis tear simultaneously before operation. The retear was mainly located in the subscapularis and partially supraspinatus tendon .Nonetheless, this patient reported improvements in pain relief and function, and a revision procedure was not performed. In the three patients with type III healing, thinning of the tendon autograft was observed, but its continuity was preserved (Figure 11). Enderwent MRI assessment Although the control group did not routinely re-examine MRI three months after surgery, however, 17 of the 23 patients underwent MRI (Figure 12).

## Discussion

Currently, no satisfactorily efficacious surgical repair techniques have been developed to treat massive irreparable PSRCTs. A high rate of postoperative retear has been reported in existing techniques, which, in some studies, could be as high as 90% (13, 14). Many factors are associated with postoperative retear, such as age, diameter of the RCTs, extent of fat infiltration and muscle atrophy, selection of surgical techniques, rehabilitation and functional exercise of the patient, and timing of resuming physical labor or activity. Such MRCTs are often accompanied by marked muscle atrophy and fat infiltration, and sometimes, superior subluxation of the humeral head, shortened acromiohumeral distance, adhesion and retraction of the torn tendon, and poor tendon quality. Therefore, extensive adhesion release is often performed during repair. However, even with extensive adhesion release, repair might not be achieved, or high tension and poor tendon quality are present despite the completion of repair, leading to a higher risk of retear. With a very high tension in the tendon, remarkable pain and poor functional recovery may be found in patients, which could also increase the risk of retear. Many strategies have been proposed to treat MIRCTs, including conservative treatment, arthroscopic debridement, transfer and fixation of the long head of biceps, partial repair of the rotator cuff, SCR, LDT, LTT, subacromial balloon spacer implantation, and reverse shoulder replacement (3, 4). For MIRCTs, partial repair is relatively widely adopted, aiming at the repair and conversion of a part of the remaining elastic rotator cuff tissue and balance of the rotator cuff force couple (15), thus easing pain and improving function and providing short-term relief of symptoms and partial improvement of function, with uncertain long-term effectiveness. Therefore, it is considered a means of alleviating the treatment (16).

SCR with fascia lata autograft was proposed by Mihata et al. as a treatment for MIRCTs, and studies have shown alleviation of shoulder pain during flexion and lifting, as well as abduction, along with marked functional improvements (17). However, fixation of the fascia lata during SCR requires at least six anchors, posing a heavy financial burden to most patients. Although inspiring outcomes have been reported after the application of the rotator cuff patch augmentation procedure to the repair of MRCTs, the patch's high cost, poor integration with the rotator cuff, slow degradation, and multiple complications seriously limit its application in clinical practice (18). Furthermore, SCR focuses on the recovery of static structural stability of the shoulder joint, stabilizing the glenohumeral joint and assisting the deltoid muscle to allow movements around the joint, although it could not secure the dynamic stability of the shoulder joint or provide a contraction force similar to that of normal rotator cuff tissues. Therefore, a normal physiological balance of the force couple could not be achieved.

Another SCR technique proposed by Boutsiadis et al. involves the coverage of the greater tubercle with the tendon of the long head of biceps, which was proven to alleviate pain and improve mobility by observational clinical studies (12). However, superior RCTs could be repaired using this technique, for patients with fat infiltration of the irreparable infraspinatus tear, effective posterosuperior tissue reconstruction could not be achieved.

For irreparable PSRCT, previous studies reported the theoretical improvement of the external rotation after LDT, rebalancing the force couple of infraspinatus and teres minor muscles, as well as its ability to depress the humeral head (19), while other studies showed only suboptimal improvement. The electromyographic response in the transferred latissimus dorsi muscle was stronger during adduction than during external rotation (20), and more rehabilitation and adaptation are required for better synergic movement of latissimus dorsi tendon (21). Retear after latissimus dorsi transfer was also reported in some patients (22, 23).

For LTT, few evidence has been published, mainly due to the insufficient length of the lower trapezius muscle, making it difficult to perform the procedure. Nonetheless, better synergic action could be achieved by the lower trapezius muscle, and its line of action was similar to that of the rotator cuff muscles (24). To compensate for the insufficient length, Achilles tendon allograft bridging and augmentation was proposed by Elhassan et al. (6–8), and studies on both open and arthroscopic procedures were conducted, with ideal results in functional reconstruction (abduction and external rotation) of the shoulder joint. Aibinder and colleagues recently reported on 41 arthroscopically assisted lower trapezius transfers. At 13 months, 37 patients (90%) had improvements in all outcome measures (25). Valenti and Werthel recently reported on the use of semitendinosus tendon autograft instead of Achilles allograft in an attempt to avoid the potential complications associated with allograft. And Constant-Murley, the simple shoulder test, the subjective shoulder value, and pain scores all improved (26).

Compared to latissimus dorsi transfer, LTT is relatively easier to duplicate, and Achilles tendon allografts are very costly and not readily available in our hospital. Compared to achilles tendon allografts, concerns regarding hamstring tendon autografts include possible insufficiency in length and width. Bridging of the lower trapezius tendon could also be conducted with autografts from three tendons, including the semitendinosus, gracilis, and peroneus longus tendons, while fixation on the humeral side could be achieved via the tunnel button plate, as proposed in a study by Zhao (27). However, too many tendons are harvested using this technique, and as many as three tunnels are created in the humerus, increasing the risk of fracture. Therefore, another technique for harvesting only semitendinosus and gracilis tendons has been developed by our team, which is already well accepted by orthopedic surgeons, and on the humeral side, surface holding and turning screw post fixation are adopted, which are easy and convenient to perform, without concerns of fracture.

After scaffolding the tendon autograft, the remaining rotator cuff tissue was stitched onto the graft. Its advantages are as follows: (1) stretching the rotator cuff to the footprint area by force is unnecessary, thus decreasing tension in repaired tissue; (2) rotator cuff stitched onto the bridging tendon, taking advantage of tendon-to-tendon healing that is theoretically superior to tendon-to-bone healing; (3) long-term functional recovery by the infraspinatus tendon due to the preservation of its remaining functions, as opposed to Bassen's technique; (4) lower tension in the repaired tissue reduces the risk of retear and improves the situation of early postoperative pain and functional disuse; and (5) fewer anchors required in the procedure, even fewer than direct repair, which requires three anchors on average, reducing the financial burden of the patients. However, its disadvantage is the uncertainty in the process of partial healing and remodeling of necrotic tissue in the tendon autograft exposed in the footprint area, whose necrosis and shedding would impair general effectiveness. Indeed, no obvious necrosis of the tendon autograft was found in the early follow-up MRI; thus, the risk of necrosis requires further investigation.

In the control group, only repairable MRCTs were available for enrollment because of the retrospective nature of this study; thus, the disease in the control group was less severe, and pain relief and functional rehabilitation should be more rapid theoretically. However, according to our follow-up data, the postoperative VAS was significantly lower in the study group than in the control group, and functional rehabilitation in the study group was also more rapid than that in the control group, as supported by the better result of CMS, UCLA Shoulder Score, ASES and in the study group. Both study and control group used the Chinese Way technique to augment the superior, which may help to improve the anterior flexion elevation. As to the function of external rotation, it is mainly accomplished by the infraspinatus and teres minor. Our result show a better improvement in the study group than control group both on the anterior flexion elevation and external rotation. This may due to a better restoration of the function of infraspinatus by the LDT with autograft technique.

**Figure 13:**
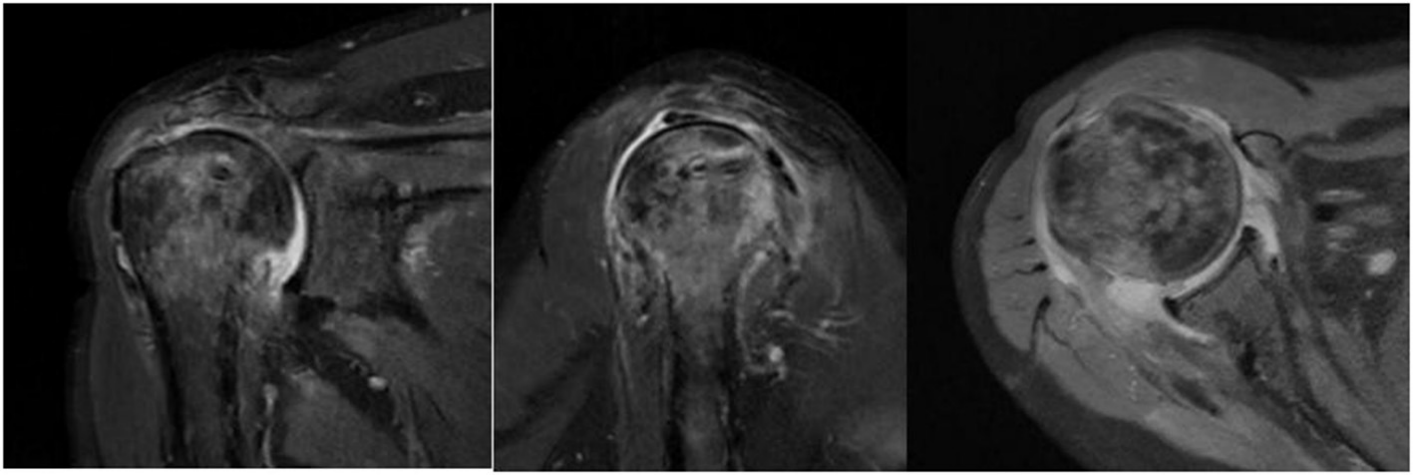
Imaging data of patients with retear in the experimental group.

The newly developed surgical technique could achieve an almost pain-free status for patients, which could persist for as long as 3 months. More obvious edema in the myofibers of the tendon was revealed by postoperative MRI in the control group, probably because higher tension in the tendon irritated the muscle belly, resulting in more edema and pain. Such pain could explain early functional disuse, while edema could also be associated with the duration of the pain and delayed functional rehabilitation, which also requires further investigation. The rapid functional rehabilitation in the study group probably resulted from lower tension, less pain, and less irritation of the muscle; however, further follow-up studies are also required to determine if their long-term outcomes are also superior to those of the control group, especially in terms of muscle strength and active range of motion. Another limitation of this study is its retrospective design, making future prospective studies necessary to confirm the strengths and weaknesses of this new surgical technique. Postoperative retear occurred in a case complicated with grade 4 subscapularis tendon injury, and the retear occurred in the subscapularis tendon and most of the supraspinatus tendon (Figure 13), suggesting that extra caution is needed when repairing massive PSRCT with severe subscapularis injury, and that additional augmentation or bridging of the SSC tendon might also be needed to prevent retear.

## Conclusion

Our study suggests that compared to conventional repair procedures, in the early postoperative period, LTT with tendon autograft could achieve better pain relief, more rapid motor functional recovery, and higher functional scores for massive irreparable PSRCTs.

## Data Availability Statement

The original contributions presented in the study are included in the article/supplementary material, further inquiries can be directed to the corresponding author.

## Ethics Statement

The studies involving human participants were reviewed and approved by the Ethics Committee of Taizhou Hospital of Zhejiang Province in China (Reference No. K20210706 approved on 12 July 2021). Written informed consent for participation was not required for this study in accordance with the national legislation and the institutional requirements. Written informed consent was not obtained from the individual(s) for the publication of any potentially identifiable images or data included in this article.

## Author Contributions

LY and XZ: conceptualization. DH and T-HT: methodology. XZ: case provider, operator, and supervision. DH and QZ: data curation. LY: formal analysis and writing—original draft preparation. XY: follow up registration. XY and T-HT: validation. T-HT: writing—review, editing, visualization, and project administration. All authors have read and agreed to the published version of the manuscript.

## Conflict of Interest

The authors declare that the research was conducted in the absence of any commercial or financial relationships that could be construed as a potential conflict of interest.

## Publisher's Note

All claims expressed in this article are solely those of the authors and do not necessarily represent those of their affiliated organizations, or those of the publisher, the editors and the reviewers. Any product that may be evaluated in this article, or claim that may be made by its manufacturer, is not guaranteed or endorsed by the publisher.
